# Analyses of Time Series InSAR Signatures for Land Cover Classification: Case Studies over Dense Forestry Areas with L-Band SAR Images

**DOI:** 10.3390/s19122830

**Published:** 2019-06-25

**Authors:** Hye-Won Yun, Jung-Rack Kim, Yun-Soo Choi, Shih-Yuan Lin

**Affiliations:** 1 Department of Geoinformatics, University of Seoul, Seoulsiripdaero 163, Dongdaemum-gu, Seoul 02504, Korea; hwyun0221@korea.kr (H.-W.Y.); kjrr001@gmail.com (J.-R.K.); choiys@uos.ac.kr (Y.-S.C.); 2 Disaster Information Research Division, National Disaster Management Research Institute, 365 Jongga-ro, Jung-gu, Ulsan 44538, Korea; 3 Department of Land Economics, National Chengchi University, No. 64, Sec. 2, Zhinan Road, Wenshan District, Taipei 116, Taiwan

**Keywords:** InSAR, time series, land cover classification

## Abstract

As demonstrated in prior studies, InSAR holds great potential for land cover classification, especially considering its wide coverage and transparency to climatic conditions. In addition to features such as backscattering coefficient and phase coherence, the temporal migration in InSAR signatures provides information that is capable of discriminating types of land cover in target area. The exploitation of InSAR signatures was expected to provide merits to trace land cover change in extensive areas; however, the extraction of suitable features from InSAR signatures was a challenging task. Combining time series amplitudes and phase coherences through linear and nonlinear compressions, we showed that the InSAR signatures could be extracted and transformed into reliable classification features for interpreting land cover types. The prototype was tested in mountainous areas that were covered with a dense vegetation canopy. It was demonstrated that InSAR time series signature analyses reliably identified land cover types and also recognized tracing of temporal land cover change. Based on the robustness of the developed scheme against the temporal noise components and the availability of advanced spatial and temporal resolution SAR data, classification of finer land cover types and identification of stable scatterers for InSAR time series techniques can be expected. The advanced spatial and temporal resolution of future SAR assets combining the scheme in this study can be applicable for various important applications including global land cover changes monitoring.

## 1. Introduction

Extracting land cover information through optical imagery analysis has been one of the primary remote sensing applications. Given recent improvements in electro-optical sensor technology with machine vision algorithms, contemporary in-orbital images with high temporal resolution have become valuable sources for effective extractions of land cover information through data mining techniques. However, despite the fact that space-borne optical sensors are capable of making frequent revisits over a target area, it is hard to acquire satisfactory images as climatic conditions often impose serious constraints on optical images. Even partial cloud cover and haze can diminish the quality of optical imagery to the extent of precluding automated data mining via machine vision techniques. Due to the limitation of optical imagery, highly time-sensitive applications should be addressed using active electromagnetic sensors, such as synthetic aperture radar (SAR), to reduce the effects in almost all weather conditions. SAR observations therefore have great potential to facilitate gauging temporal features.

Signatures derived from interferometric SAR (InSAR) processing, including phase coherence and amplitude, provide valuable information of the properties of scanned surfaces. There have been many applications of InSAR signatures for land cover classification, for example biomass forecasting [1,2], observations of forests [3], and study of arid land surface characteristics [4]. After Wegmüller et al. [5] first recognized the importance of InSAR products for land cover classification, Strozzi et al. [6] constructed a land cover map using European Remote Sensing Satellite (ERS) InSAR products. To exploit the short repeat-pass interval, which helps to maintain the sensitivity of phase coherence to land cover type, Santoro et al. [7,8] employed ERS and Environmental Satellite (ENVISAT) interferometry to extract mining information about vegetation in a land cover investigation. Based on the studies listed above, it is known that the acquisition of reflection from long wavelength electromagnetic energy provides useful tools for understanding the characteristics of scanned surface.

Since previous research has demonstrated the potential of InSAR for land cover mapping, we focused on an integration of InSAR signatures to provide a means of constructing the feature space necessary to classify land cover types. Together with the advantage of high-temporal resolution, a new time series analysis was employed to provide accurate land cover information, particularly to determine the most effective approach for building the feature space. The classification scheme proposed in this study centers on using the packet assembly of InSAR signature times series and its linear and nonlinear transformations. To demonstrate the performance of the developed classification scheme, we assessed the results through the comparison with products derived from the MODerate resolution Imaging Spectroradiometer (MODIS), Earth Observing-1 (EO-1) Advanced Land imager (ALi), and Landsat 8 imagery. The InSAR products and methodology for classification are introduced in Section 2 and Section 3, respectively. The test sites and the corresponding results are then reported in Section 4 and Section 5.

## 2. Background of InSAR Land Cover Classification

The backscattering coefficients derived from single amplitude image and phase coherence of the interferometric pair are both useful parameters to achieve successful image classification [9]. The phase coherence of two conjugated complex SAR pixels can be expressed as follows:

(1) $$coh=\frac{\sum_{i=1}^{N}S_{Mi}S_{Si}^{*}e^{j\emptyset \left(i\right)}}{\sqrt{\sum_{i=1}^{N}{\left|S_{Mi}\right|}^{2}}\sqrt{\sum_{i=1}^{N}{\left|S_{Si}\right|}^{2}}}$$

where *S_Mi_* and $S_{Si}^{*}$ are the complex conjugated signals of master and slave SAR images, *N* is the total number of signals within the estimated window, and *Ø*(*i*) is the phase of the *i*th signal of the images.

Given that phase coherence largely depends on geometric variation in reflectors, the reflection of radar waves over barren fields produces high phase coherence. On the other hand, forestry coverage is an example of a reflector generating low phase coherence. In addition, the SAR amplitude and backscattering coefficients established through radiometric processing also possess useful information associated with the physical and geometric properties of scanned materials, such as permittivity and roughness. As each type of land cover has inherent phase coherence and backscattering coefficient, the potential of interferometric signatures for the identification of land cover types has been noted ever since initial SAR applications and was firstly explored by Wegmuller and Werner [3]. In their research, three feature spaces were established for the exploitation of InSAR signatures: (1) phase coherence; (2) backscattering coefficients; (3) the difference in backscattering coefficients. This strategy was implemented based on phase coherence changes over different types of land cover. For example, there is a strong temporal correlation over bare fields and weak phase correlation over vegetated canopy. Characteristics of SAR backscattering coefficients are also useful in compensating phase coherence for land cover classification. These characteristics include weak signatures in water, weak to medium signatures according to the density of vegetation, and strong signatures in artificial structures. Color composite images with phase coherence in the Red (R) channel, backscattering coefficient in the Green (G) channel, and the generated backscattering coefficient difference in the Blue (B) channel regardless of polarization mode has been employed to investigate land cover types visually [3,4]. However, the features in RGB SAR signature combinations from observations taken at different times are inconsistent and hence limit the establishment of quantitative land cover measurements. Therefore, it is necessary to consider alternative SAR data combination for efficient land cover extraction.

As the properties of physical scatters and temporal variations are correlated, not only single image, SAR signatures extracted from time series of InSAR interferograms are also valuable. For example, a vegetated canopy shows significant changes in phase coherence and amplitude over time because of growth and time-specific events such as wind direction and changes in moisture. In contrast, bare fields and artificial structures maintain a relatively constant phase coherence and amplitude over time. As such correlation is clear and can be observed in time series InSAR phase coherence and backscattering coefficients, it becomes sensible to introduce sequence of InSAR pairs. Moreover, since the stacked time series of InSAR interferograms and their interpolations, such as small baseline subsets [10] and permanent scatterers [11], have proven effective to handle error components, applications of continuous InSAR image pair acquisitions over single target areas has become a common approach. Due to the advantages described above, utilization of InSAR time series is preferable for land cover analysis and is therefore applied in this study.

## 3. Methodology

With the introduction of InSAR time series, we aimed to develop an enhanced method to analyze a collection of a series of InSAR pairs, which were indicated as a packet (refer to Section 4 for detailed concept and implementation), over a given time domain. As reported by Bruzzone et al. [12], extracting the statistics such as mean values and standard deviations for phase coherence and amplitude vectors is a basic way to build features from time series InSAR products. However, the random noise commonly associated with phase coherence and amplitude vectors of InSAR pairs can significantly skew the means and standard deviations of these characteristics. Moreover, weak correlated InSAR pairs contribute equally to these statistics, potentially resulting in unexpected feature values. To address these issues, we applied dimensional compression through principal component analysis (PCA) [13]. The time series was transformed through PCA and the effects of weak correlated InSAR signatures were excluded. From the preliminary experiments, we observed that the first principle component usually included constant values for phase coherence and amplitude. The values were used as the replacements for the means of signatures. The second PC normally included values that varied widely. Once the random noise components were excluded, values in second PC were used as an effective indication for temporal variation. As higher order components of PCA were noisy in packets, these components were not considered in establishing surface features.

Through the processing, we also found that the noise component in the SAR signatures significantly impaired the capability of classification scheme. The errors were due primarily to inconsistent phase coherence caused by associated weather factors, such as wind and moisture change, and mis-coregistration of SAR images. Even with PCA transformation, a large number of errors in InSAR signatures were not screened out in high-order components and degraded the classification efficiency. To improve the performance, we further introduced kernel PCA (k-PCA) to replace standard PCA. Nonlinear transformations through k-PCA reduced errors in SAR signatures and constructed more robust feature spaces. This study used a nonlinear kernel matrix instead of the Gram matrix introduced for PCA as proposed by Scholkopf et al. [14]. The kernel matrix is expressed as:

(2) $$K=k\left(x,y\right)=\Phi {\left(x\right)}^{T}\Phi \left(y\right)$$

where *Φ(x)* is a nonlinear function of feature vector *x*.

The projection of feature vector *x* on a principle axis, *w_i_*, is represented as:

(3) $$w_{i}^{T}\Phi \left(x\right)={\left(\sum_{i=1}^{N}a_{i}\Phi \left(x\right)\right)}^{T}\Phi \left(x\right)$$

The calculation and normalization can be completed by the following eigenvector equation:

(4) Nλa=Ka

where *N* is the number of observations and *λ* and *a* are the eigenvalues and eigenvectors of *K*, respectively. This study employed the following Gaussian kernel matrix:

(5) $$k\left(x,y\right)=\exp\left(-||\Phi \left(x\right)-\Phi {\left(y\right)||}^{2}/2\sigma^{2}\right)$$

where *σ* represents the standard deviation. As Mika et al. [15] stated, this type of processing is useful for removing extraneous noise from the data and for constructing new feature spaces that are representative of the nonlinear component of observations. These steps are essential for rigorous analyses due to the complexity of InSAR signatures over different types of land cover particularly in phase coherence. Employment of phase coherences as the signatures for land cover classification is the variation of temporal incoherence issue. The total coherences can be decomposed as:

(6) $$Coh=Coh_{thermal\;}Coh_{spatial\;}Coh_{temporal\;}$$

The thermal coherence *Coh_thermal_* and the spatial coherence *Coh_spatial_* are respectively expressed as:

(7) $$Coh_{thermal}=\frac{1}{1+SNR^{-1}},\;Coh_{spatial}=1-\frac{2\left|B\right|R_{y}cos^{2}\left(\theta -\alpha \right)}{\lambda \rho }$$

where *B* is the perpendicular baseline, *R_y_* is range resolution, *θ* is the incidence angle, *λ* is the wavelength, *α* is the local surface slope and *ρ* is the distance between sensor and target image [16]. The thermal and spatial coherence are extractable from system and orbital characteristics such as by perpendicular baseline and can be compensated. Moreover, as the thermal and spatial coherences are close to unique values, their effects to land cover classifications are minor.

Temporal coherence is a complicated issue. Lee et al. [17] described the pattern of temporal coherence as below:

(8) $$Coh_{thermal}=\exp\left(-C_{t}\Delta T\right)$$

where *C_t_* is the decay constant of temporal coherence and Δ*T* is the time period of InSAR pair observation. We assumed that the decay patterns of L-band phase coherences correspond to the land cover types. Hence we modified the model of temporal coherence as listed below:

(9) $$Coh_{thermal}=C_{1}+C_{2}\exp\left(-C_{t}\Delta T\right)$$

where *C*_1_ is the constant to converging value of temporal coherences and *C*_2_ is the depending parameter of the characteristics of land cover types. It was expected that the temporal observation of phase coherence in time series could be useful for land cover classification based on the characterized model of temporal coherence. This assumption was reviewed using the classified land cover in Section 5.

Although k-PCA is highly effective at suppressing random noise and accounting for the nonlinearity of signatures, there are still mixed components of newly constructed InSAR features, resulting in misclassifications between land cover types. Therefore, an advanced classification scheme that manipulates mixed InSAR signatures, rather than relying on the conventional classification algorithms such as maximum likelihood (ML), was introduced. Support vector machine (SVM) analysis is expected to address classification errors in this case because it maximizes the extent to which spaces can be subdivided, using hyperplanes as distinguishing functions. This methodology was introduced by Cortes and Vapnik [18]. The hyperplane approach of SVM effectively addresses the ambiguous nature of some InSAR signatures through nonlinear classification. SVM is also based on a kernel operation in which a vector of feature spaces is transformed into hyperplanes as follows:

(10) $${\left(\sum_{i=1}^{N}a_{i}\;\Phi \left(x\right)\right)}^{T}\Phi \left(x\right)=Constant$$

Equation (10) is used to distinguish and measure a single vector. By introducing nonlinear operations into transformation *Φ*(*x*), the SVM can provide a robust method for classifying unstable features, such as InSAR signatures. After all, the whole processor we proposed was composed of the k-PCA of backscattering coefficients and phase coherences respectively, and the SVM classifier of newly constructed features by nonlinear transformations together with the pre-processors of speckle filtering and radar shadow masking. The k-PCA plus SVM classification schemes for accommodating the nonlinearity of feature space were conducted. To assess the performance of k-PCA plus SVM scheme, traditional PCA plus ML processor was also implemented. The results derived from two processors were analyzed and reported in Section 5, showing the classification results and vegetation indices from optical image.

It was noted that for all constructed features, classification algorithms were tested using training vectors measured by 10 m resolution EO-1 ALI images and partially, i.e., for the eastern area, by the Google Earth historical image archive which was taken by Digital Globe IKONOS with 0.8 m panchromatic and 3.2 m multispectral resolution. It was clear that such training vector definitions using the secondary information would downgrade the classification accuracy. However, as ground surveying data over Mt. Baekdu were not available, the use of secondary information for the training vector definition was unavoidable.

## 4. Test Sites and Data Description

We selected two test sites with different land cover types to investigate the performance of the proposed classification scheme. The first test area is around Mountain Baekdu (Mt. Baekdu, also known as Mt. Changbai in Chinese), a volcano located on the border between North Korea and China. The site is covered by dense vegetation with minor settlements and bare fields. In order to assess the potential risk caused by various natural disasters including floods, landslides, forest fires and volcanic activities, the land cover map over Mt. Baekdu is essential. However, due to local political tensions, reliable land cover datasets were prevented from being published. Up to now, only a limited number of precedent studies for determining land cover over Mt. Baekdu have been conducted with space-borne optical imagery. For instance, Liu et al. [19] employing Landsat Thematic Mapper (TM) images over Mt. Baekdu showed the dependency of tree species on the altitude. A study conducted by Park et al. [20] analyzed MODIS normalized difference vegetation index (NDVI) change over the China and North Korea border together with land cover types. In order to achieve a comprehensive understanding of environment over Mt. Baekdu, we conducted a time series land cover monitoring based on InSAR analysis in the first test site and evaluated its performance.

Since SAR signatures are useful to define land cover types, it is necessary to consider the appropriate SAR wavelength. As C-band ERS and ENVISAT ASAR image data were constrained by the physical penetration depth, their InSAR signatures are primarily reflected over vegetated canopies. Thus, the C-band wave is highly sensitive to changes in moisture and wind direction over time [4]. This indicates that, considering both the expected weak phase coherences over the vegetated and the steep sloped areas, C-band SAR images may not be suitable for investigating land cover over dense forest in the test sites. ENVISAT ASAR data was therefore excluded from the pool of potential data sources. In contrast, due to the characteristics of long wavelength, SAR images taken by Phased Array type L-band Synthetic Aperture Radar (PALSAR) installed on the Advanced Land Observation Satellite (ALOS) were expected to be ideal data sources as they were capable of producing high phase coherence over highly vegetated areas. For instance, ALOS PALSAR images pairs in our study demonstrated 2–3 times higher phase coherence over the area with Enhanced Vegetation Index (EVI) > 0.2 compared to ENVISAT ASAR case of corresponding temporal baseline. Although PALSAR images also have difficulty to effectively distinguish certain land cover types, such as grass and crops, L-band SAR signatures are useful tools to discriminate open/mixed forests, which is the main land cover types in our test sites. Time series PALSAR observations are also helpful to define seasonal effects occurred in the field. Therefore PALSAR images are applied, where a total of 12 ALOS PALSAR Fine Beam Mode Single Polarization (FBS) and Fine Beam Mode Dual Polarization (FBD) images over Mt. Baekdu acquired during 2009–2011 were employed in this study. The details of the PALSAR images used are listed in Table 1.

The second test site was in Uljin located in the eastern Korea, where dense forests are widely populated. Since few residential and cultivation areas are also distributed here, it can be used for the assessment of an algorithm employed for classifying minor land cover types. Over Uljin target areas, nine ALOS PALSAR FBS and FBD images were employed and the details are listed in Table 2. As ALOS PALSAR images were used in this study, considering the characteristics of L-band data, land cover type definition was introduced within the context of targeted geometric properties that were defined with respect to the standing heights and densities of vegetated materials. A total of five land cover types were assigned to Mt. Baekdu, including bare fields, open/mixed forests, conifer forests, water bodies and shrubs/grasses. While for the case of the Uljin area, the artificial structures were introduced instead of a bare field considering the land cover features of the target area. Radar-shadowed areas, which sometimes are misclassified, were predefined, according to conditions existing during the acquisition of SAR images. These areas were then masked off in each of the processing stages.

**Figure 1:**
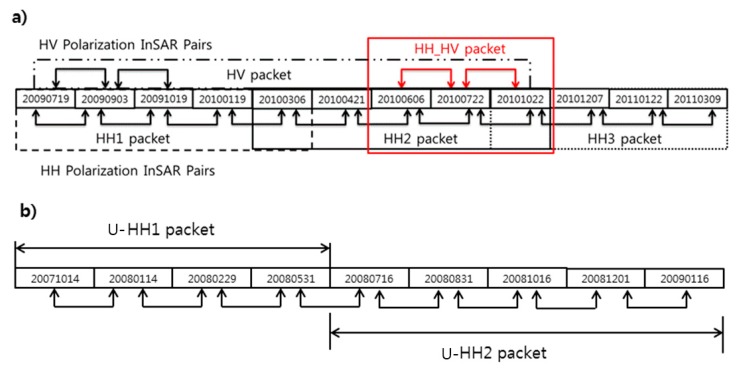
Employed InSAR time sequence pairs and the packet compositions in Mt. Baekdu (**a**) and Uljin (**b**).

To evaluate the InSAR packet approach, a set of InSAR packets were constructed considering adjacency of InSAR pair and seasonal coverages. Figure 1 illustrates the composition of packets in this study, including HH1 (2009/07/19–2010/03/06), HH2 (2010/03/06–2010/10/22), HH3 (2010/10/22–2011/03/09) and HH_HV (2010/06/06–2010/10/22) packets in Mt. Baekdu, while HH1 (2007/10/14–2008/05/31) and HH2 (2008/07/16–2009/01/16) in Uljin. Regarding registration accuracy of image pairs within InSAR packets, all data sets were crossly registered by the InSAR processor up to sub-pixel accuracy.

## 5. Processing Results and Discussion

**Figure 2:**
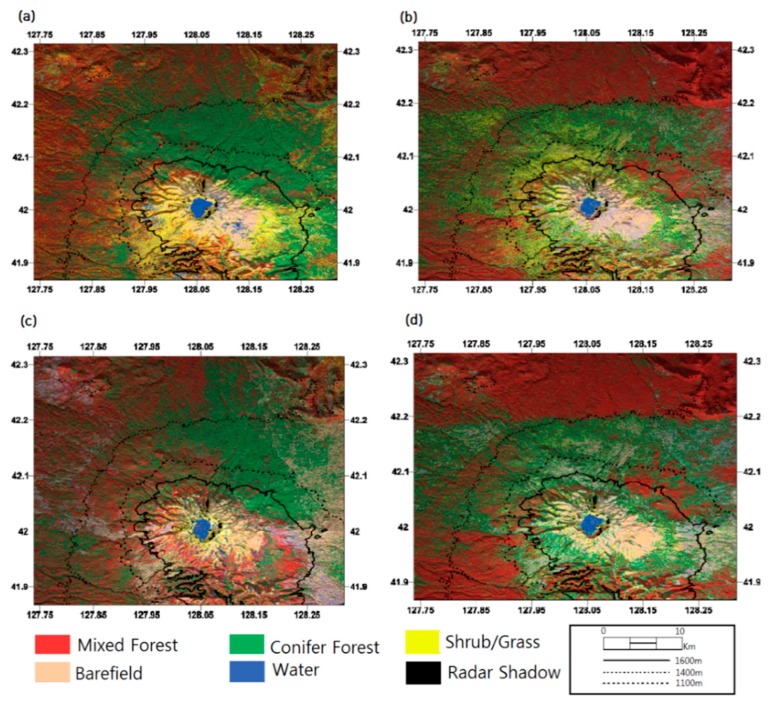
The land cover classifications with PCA/ML over Mt. Baekdu from (**a**) HH1 packet during 2009/07–2010/03; (**b**) HH2 packet during 2010/03–2010/10; (**c**) HH3 packet during 2010/10–2011/03; and (**d**) HH-HV packet during 2010/06–2010/10.

The initial classification results derived from conventional ML classifiers and InSAR features set by PCA analysis are shown in Figure 2, in which classification maps of HH1–HH3 packets and a hybrid packet are illustrated.

**Figure 3:**
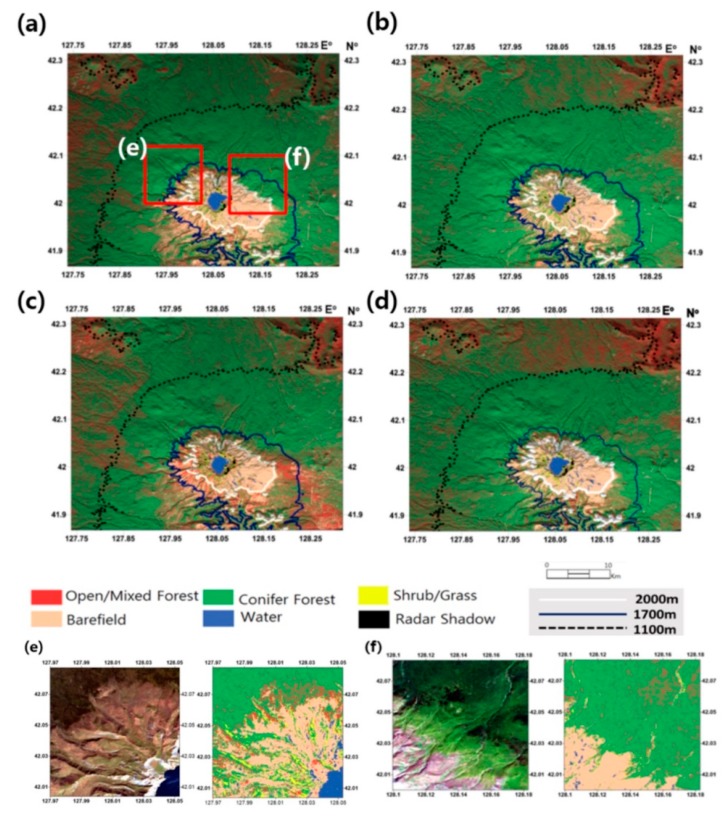
The land cover classifications with k-PCA plus SVM scheme over Mt. Baekdu from (**a**) HH1 packet during 2009/07–2010/03; (**b**) HH2 packet during 2010/03–2010/10; (**c**) HH3 packet during 2010/10–2011/03; (**d**) HH-HV packet during 2010/06–2010/10, and (**e**–**f**) LANDSAT sub-images and corresponding HH1 packet classifications.

The InSAR classification maps generated through k-PCA plus SVM processor are shown in Figure 3. Due to a lack of ground truth for verifying classification results, we alternatively found altitudinal distribution map of vegetation species originated by the volcanic investigations of Mt. Baekdu as the reference data. Zhao [21] identified that the normal altitudinal dependency of tree species from conifer to deciduous trees is broken in the south-eastern flanks of Mt. Baekdu, which is due to directional intensive tephra covering. Subsequent studies conducted by He et al. [22], Dai et al. [23,24], and Park et al. [20] continued to identify the tree distributions. The altidudial tree distribution over Mt. Baekdu from their studies included: (1) tundra over 2000 m altitudinal line; (2) birch dominated forest between 1700–2000 m altitudinal line; (3) spruce fir and birch mixed conifer forest between 1100–1700 m altitudinal line; (4) pine and broad-leaved mixed forest between 740–1100 m. As the findings were commonly supported by most of the literatures, the altitudinal dependency was used as the reference data to assess the classification accuracy. In order to compare with our observations, we mapped our land cover classes to their altidudial tree species dependences based on following principles: (1) tundra to barefield; (2) birch dominated forest together spruce fir to conifer forest and (3) pine and broad-leaved mixed forest to open/mixed forest. It was found that the altitudinal tree species transition lines (1100 m, 1700 m and 2000 m) presented in Figure 3 fit well with our land cover classification types, especially transition between bare field and conifer, and conifer and open/mixed forest.

Moreover, it was observed that the altitudinal dependency in the south-eastern flanks was broken in the classification results as Zhao [21] assigned (refer to dotted ellipse in Figure 3). While the classification demonstrated in Figure 2 shows that open/mixed forests extend up to an altitude of 1600 m and conifers rarely distributed. The results disagreed with the altitudinal distribution summarized above, indicating a PCA feature extraction scheme failed to provide reliable signatures to support classification. Therefore we concluded that the land cover classification scheme using k-PCA and SVM retained better efficiency and identified the land cover types well, especially vegetation distributions.

Overall, the classifications from all packets in Figure 3 produced similar results, in which different distributions of trees in open/mixed forests between HH1 and HH3 are found over the target area. The open/mixed forest coverage was minimal in the classification from the HH2 packet because the InSAR images were acquired during spring and summer. Open/mixed forest coverage was more extensive in classification results from the HH1 packet that included InSAR data from the fall and winter. InSAR images for HH3 were acquired in the winter and early spring. The images were affected by seasonal factors such as snow cover and fallen leaves, therefore, the classification results from HH3 show limited distribution of open/mixed forest. It should be noted that the land covers classified by the HH-HV packets did coincide well with the land cover classified by the HH packet signatures. The compilations of different polarizations in HH-HV packets was performed to produce an enhanced feature space to effectively distinguish forest cover types. The conifer tree lines for all packet combinations showed agreement with the 1700 m altitude, presumably owing to the consistency of InSAR signatures, covering all seasons, from conifers.

**Figure 4:**
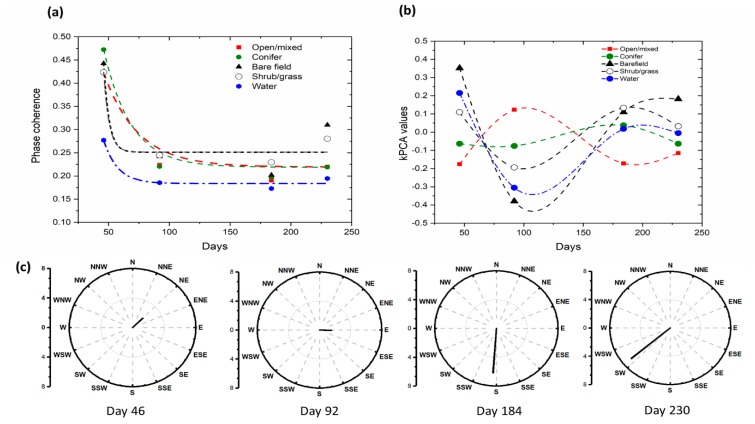
(**a**) The temporal phase coherence migrations of land cover types in HH2 packet; (**b**) The temporal patterns of transformed k-PCA components; (**c**) Wind components of pairs in HH2 packet, those were extracted from the ECMWF model.

Based on the classification outputs, we also investigated the effectiveness of k-PCA, PCA and original phase coherence time series as the phase coherence signature involving the temporal coherence issues as described in the Section 3. The temporal decaying pattern of phase coherence on land cover type followed the characterized models but demonstrated somewhat similar patterns especially over some vegetation types such as conifer-to-open/mixed forest case (see Figure 4a). In addition, temporal phase coherence has quite dependences on the weather condition at the times of image acquisitions. For instance, compared to the European Centre for Medium-Range Weather Forecasts (ECMWF) wind information demonstrated in Figure 4c, the deviations of third and fourth InSAR pairs from decaying model might associate with the wind effects on vegetation leaves as described in Wegmuller and Werner [3]. As also stated in Wegmuller and Werner’s analysis, the moisture changes in vegetation canopy and soil induced by the precipitation can produced significant effects in phase coherence as the permeability of target electromagnetic matter become inconsistent interacting with wind speed and direction. However, due to the following two reasons: (1) the precipitation effects on the phase coherence over the target area was mainly produced by the local turbulences which is not predicted by ECMWF [25]; (2) the mechanism of phase coherence change by the precipitation is largely complicated due to the interacting with the wind, we only analyzed the inter-relationship between phase coherence considering that wind component may imply some effects of local precipitation that cannot be accurately investigated independently. Those factors caused uncertainties for the establishment of land cover classification scheme using time series phase coherence. On the other hand, phase coherences with k-PCA transformations produced well distinguished temporal signatures as shown in Figure 4b. It is noted that the spline lines fitting k-PCA values of land cover types implied that the transformed temporal migration of k-PCA values consists better discriminated signatures but less influenced by wind environment. On the other hand, eigenvalue analysis of PCA transformation presented the first components of PCA transformation retained only 60–65% of the total variance; thus non-linear k-PCA is the better approaches to create proper features from highly scattered signatures. This advantage proved that the k-PCA analysis we proposed to generate transformed signatures was appropriate to be used for an advanced classification scheme, especially a new feature space construction method, to efficiently extract land cover data from SAR signatures.

The effectiveness of k-PCA/PCA of phase coherence packets for establish the proper signature was assessed by the Jeffreys-Matusita (J-M) distance [5]. It is represented below as the metric:

(11) $$J_{ij}=\sqrt{2\left(1-e^{-B}\right)},\;B=\frac{1}{8}{\left(\mu_{i}-\mu_{j}\right)}^{t}{\left\{\frac{M_{i}+M_{j}}{2}\right\}}^{-1}\left(\mu_{i}-\mu_{j}\right)+\frac{1}{2}\ln\left\{\frac{\left|\left(M_{i}+M_{j}\right)/2\right|}{{\left|M_{i}\right|}^{1/2}{\left|M_{j}\right|}^{1/2}}\right\}$$

where *M_i_* is the covariance matrix of class *i*, *µ_i_* is the mean values of class *I* and |*M_i_*| is the determinant of the covariance matrix of class *i*. *I_ij_* is the *J-M* distance, for which high distance indicates high efficiency.

**Figure 5:**
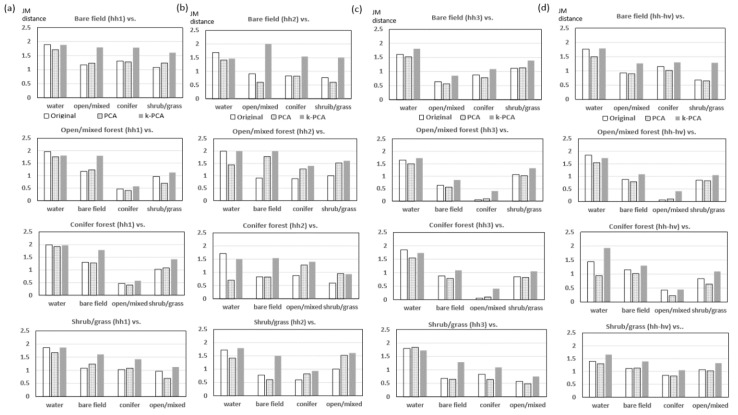
Land cover separabilities by Jeffreys-Matusita distances. It measured over simple phase coherence time series, PCA components (1 to 3) and k-PCA of HH1, HH2 HH3 and HH_HV packets.

Jeffreys-Matusita distances for all land cover combinations using phase coherence time series, PCA and k-PCA components were extracted and shown in Figure 5.

It is demonstrated that the efficiency of k-PCA approach to establish better signature for land cover classification compared to the simple phase coherence time series. Firstly, it was noted that the last component of the conventional PCA signature was excluded, thus three major components among all four signatures failed to provide better separability compared to the original phase coherence time series. It could be inferred that various noise component described in Section 3 was not well discriminated in the land cover signatures. On the contrary, the k-PCA provided better separability for all land cover combinations except water, which was expected due to the fact that the water surface induced always the lowest phase coherence. For all other land cover combinations, the separabilities of k-PCA approach were constantly higher even the case of the HH2 packet with the shorter duration. It means k-PCA approach properly addressed the seasonable phase coherence variation.

**Figure 6:**
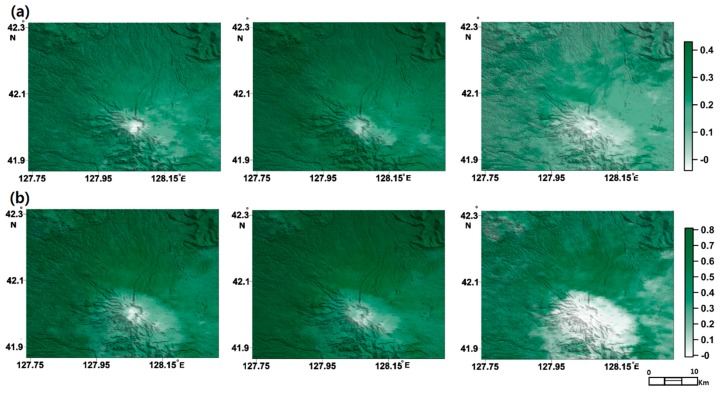
NDVI (**a**) and EVI (**b**) map over Mt. Baekdu target area from MODIS land products in HH1 period (**left**), HH2 (**middle**) and HH3 (**right**).

Evaluating quantitatively the classification results for the Mt. Baekdu test area was also difficult since no reliable source of information about the actual composition of land cover in the area is available. We firstly assessed the accuracy of our results by comparing MODIS land products (e.g., vegetation indexes) with the correlation between vegetation classes and altitude. MODIS EVI and NDVI maps extracted using the temporal average of MODIS MOD 13 products corresponding to InSAR packet periods (Figure 6) show that the EVI and NDVI in the target area became maximized in the HH2 packet period corresponding to summer season and minimized in the HH3 packet period which was taken during winter-spring season. It was found that the classification results using the InSAR signature packets was capable of capturing temporal changes in vegetation density. EVI distributions (Figure 6b), particularly those corresponding to the HH3 packet period, are similar to extracted land cover distributions, perhaps due to the less saturated nature of EVI.

**Figure 7:**
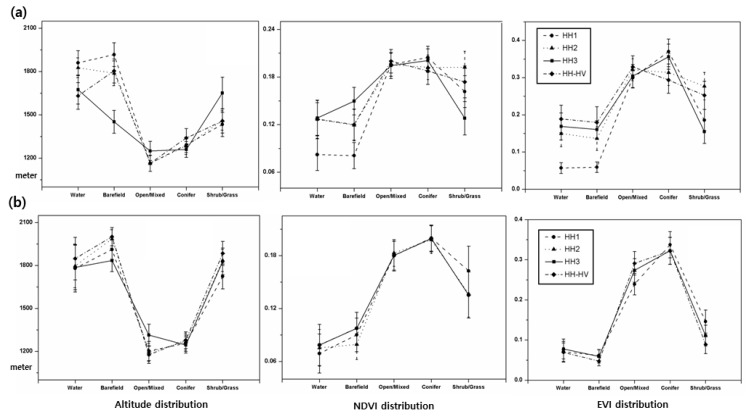
MODIS vegetation indexes and altitudes within classified land cover types by centered PCA (**a**) and k-PCA (**b**) schemes over Mt. Baekdu with altitude, NDVI and EVI.

As illustrated in Figure 7, we performed additional analysis by computing the mean vegetation index and altitude for each type of land cover. Results confirmed that land cover types depended on vegetation indexes and altitude as two distinguishing characteristics of target area environments. In Figure 7a, it is shown that PCA analysis produces less consistent values of mean vegetation indices for each land cover class than k-PCA does, indicating that k-PCA performs better classification. However, per k-PCA results (Figure 7b), the altitudes and vegetation indexes for each land cover class are clearly consistent and possess only small deviations. For instance, low NDVI and EVI values for shrub cover on high-altitude mountain summits agree with the map presented in Figure 6b. The results also demonstrated that NDVI values for low-altitude open forests were less than NDVI values for conifer forest coverage. According to results shown in Figure 7, conifer forest coverage naturally returns the highest vegetation index values, which demonstrates the robustness of the approach using InSAR time series analysis.

Detailed information about the optical images employed for the inter-comparison is given in Table 3. The temporal adjacency to the employed InSAR packet and then cloud fraction was firstly considered for the optical image selection. The subpixel registration accuracy compared to InSAR image were checked using manually measured control points and SVM classification were applied afterward. In the form of a confusion matrix, Table 4 provides the results of quantitative comparison against the classification implemented based on Landsat 8 optical images over Mt. Baekdu. The optical image acquisition time was temporally similar to the HH2 InSAR time series. Other metrics to assess classification accuracy in Table 3 were calculated as following notations [26]:

(12) $$\mathrm{Overall}\;\mathrm{accuracy}\;\left(\mathrm{OV}\right)=\frac{TP+TN}{T},\;\mathrm{Kappa}\;\mathrm{coefficient}\;\left(\mathrm{KC}\right)=\frac{T\left(TP+TN\right)-S}{T^{2}-S}$$

where *TP* is the number of corrected extract class, *TN* is the number of corrected rejected class, *FN* is the number of undetected class, *FP* the number of incorrected extracted class, *T* is total number of pixel in test and *S* is *(TP + FP)(TP + FN) + (FN + TN)(FP + TN).*

It is observed that the k-PCA–SVM scheme performed better than PCA-ML in terms of metric of confusion matrix. Comparing optical image classification demonstrated relatively good agreement, except for the shrub and grass cases. Since there is no assurance for the classification accuracy of LANDSAT-8, the values only demonstrated the relatively high reliability of the InSAR packets approach. The low coincidence in the shrub and grass classes might have originated from the full penetrability of InSAR signatures over shrub and grass areas, given their short height.

Based on the results demonstrated over Mt. Baekdu, all packets clearly delineated reasonable boundaries between shrubs, open/mixed forests and conifer forests. Moreover, after comparing with the classification results from the PCA analyses, the performance of k-PCA-SVM scheme with time series packets was significantly improved in accuracy and efficiency.

**Figure 8:**
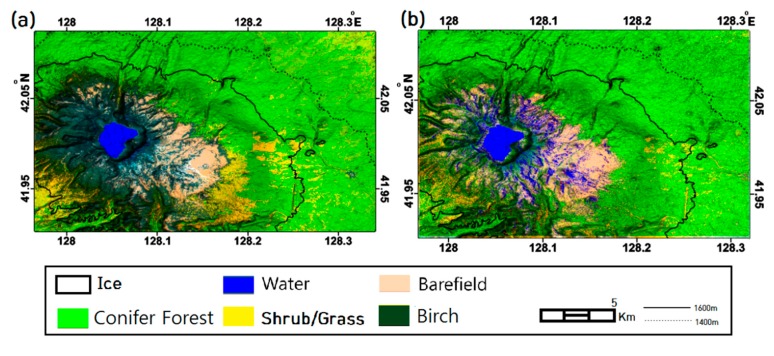
The detailed land cover classifications over Mt. Baekdu from EO-1 ALi optical image SVM processing (**a**) and HH1 packet with k-PCA plus SVM analysis (**b**).

With the improved performance, it was found that few errors occurred in the land cover classification maps. Firstly, due to radar characteristics, roads and airfields, which comprise only very small portions of the target area, could not be distinguished from barefields. Shrubs/grasses and cultivations could not be differentiated because of the similarity between their standing heights and the deep penetration of the L-band waves. Secondly, the land cover classification maps respectively derived based on the same training vectors and land cover types from SAR and optical EO-1 ALi image were compared (as shown in Figure 8). The conifer and birch forests seem to have been classified. However, as we were unable to find SAR and optical images covered similar temporal period, which becomes the limitation of this approach, their distributions in the optical and InSAR time series schemes are not very similar. For the same reason, snow cover is identified in the InSAR time series scheme as a water body. Thirdly, decorrelation over steep topography [27], together with snow cover in the winter, might be the reasons resulting in classification errors in summit areas. Since phase decomposition was capable of eliminating such errors as reported by Wang et al. [28], the introduction of phase decomposition will be further introduced in future improvement.

Considering the dependences on the weather condition and consequent effect of land cover classification, L-band SAR is less sensitive for temporal phase coherence change [5]. To investigate the robustness of L-band phase coherence signature, we tested the L-band phase coherence land cover classification scheme over Uljin, the second test site where the orographic effects caused the seasonable wind and precipitation changes [29]. It usually presented significant draught in spring to summer season and heavy precipitation in wintertime together with corresponding wind directional change.

As shown in Figure 1, the packets covered the winter and summer seasons, it is known that the phase coherence deviation from temporal coherence model is significant due to the orographic effects as described.

**Figure 9:**
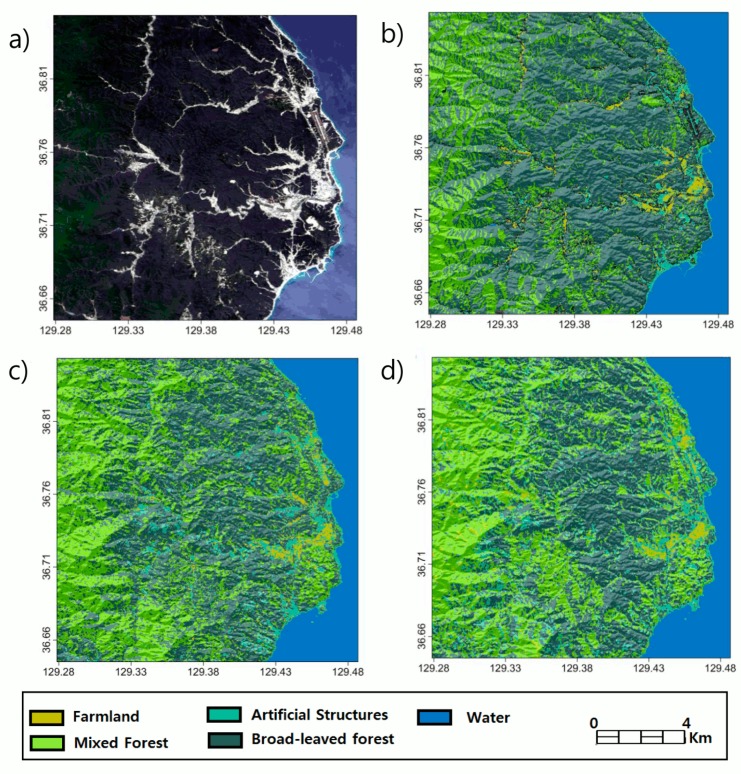
Landsat 8 visual channel image (**a**) and the land cover classification map (**b**) over Uljin target area. (**c**) and (**d**) respectively illustrate the land cover classifications conducted using k-PCA plus SVM scheme based on HH1 packet during 2009/07–2010/03 and HH2 packet during 2010/10–2011/03.

As shown in the Figure 9a contrast image, it mostly covered dense forest where the EVI is higher than 0.3 and mostly classified as mixed and broadleaf forest area in Figure 9b with Landsat 8 images. Figure 9c,d show the k-PCA classification results of InSAR signatures in Uljin. Notably, we employed two HH packets and also tested their ability to discriminate artificial structures, which were less distributed in Mt. Baekdu case. The discrepancy between U-HH1 (Figure 9c) and U-HH2 (Figure 9) classification occurred in forest areas was explained by seasonal effects. Moreover, the distribution of artificial structures in U-HH2 classification was less than that of U-HH1, and the resolution limit of the ALOS PALSAR product could have resulted in the relatively different classification accuracy in spatially mixed land cover types.

Table 5 shows the results of classification evaluation over Uljin compared with the Landsat 8 image classification. The low accuracy of urban and residential areas might have originated from the highly mixed land cover types over farmlands, where artificial structures and crops are closely populated and thus cannot be discriminated well by the low resolution of ALOS PALSAR.

Overall, the tree distribution by land cover type over Mt. Baekdu agreed well with the results of the precedent studies. The extracted altitude dependency in particular confirmed the accuracy and effectiveness of our classification algorithm for investigating vegetation. However, the InSAR packet approach we proposed could not classify specific types of land cover (e.g., roads and cultivation areas) as the image resolution is not high enough to produce characteristic InSAR signatures. To address this, finer-resolution L-band SAR images (e.g., ALOS PALSAR-2 and future NASA-ISRO Synthetic Aperture Radar (NISAR)) should be used to classify more detailed types of land cover. Regarding the data employed to the SAR packet approach, although it is difficult to achieve reliable land cover classification over the environment covered by forest, water and snow using C-band SAR characteristics, we are expecting that short temporal baseline C-band InSAR, such as Sentinel-1, will be applicable for the land cover classification in flat built-up area where the main aim of the classification scheme is to discriminate artificial structures and partial vegetation.

## 6. Conclusions

Since repeat-pass InSAR observations have frequently been used to assess natural and anthropogenic hazards, including landslides, seismic and volcanic activities, we investigated whether a series of signatures from repeat-pass InSAR processing (e.g., amplitude and phase coherence) can be of help to extract land cover data. Considering signatures from single InSAR pairs are sensitive to temporal effects to produce reliable land cover classifications, InSAR time series extracted from repeat-pass InSAR processing can be useful for developing consistent classification schemes. We therefore implemented advanced approaches with k-PCA and SVM to extract reliable land cover classifications from InSAR time series signatures and applied them to Mt. Baekdu and Uljin test areas, both of which are characterized by a mixture of diverse vegetation, bare fields, and bodies of water. Together with phase coherence, amplitude, and differences in amplitude between passes, k-PCA plus SVM approaches extracted consistent land cover classes and even captured temporal variation over vegetated coverage.

Although classification errors originated from phase coherence and despite radar illumination and weakly registered areas, we clearly demonstrated the effectiveness of L-band InSAR time series signatures in developing comprehensive land cover classification schemes. Once InSAR time series classification schemes are fully implemented, it will be beneficial to apply them to high-resolution C- and X-band InSAR series in order to develop a refined land cover map over built-up areas where the decorrelation issues are insignificant compared to the target areas in this study. Given that the decommissioning of ENVISAT ASAR, ERS, and ALOS PALSAR, we need other resources to continuously update repeat-pass InSAR images. Now, the next generation of SAR fleets have already been built and include orbital insertions of ALOS-2 PALSAR, Sentinel-1, and a few high-resolution X-band SAR satellites. Upon installing the equipment, land cover classification schemes from InSAR packets will be able to conduct highly profitable environmental monitoring, even without further acquisition of optical images from target areas. Especially, it is worth noting that the newly established spaceborne sensor such as Global Ecosystem Dynamics Investigation (GEDI) and Ice, Cloud and land Elevation Satellite (ICESAT) 2, which are capable to produce active measurements over vegetation can provide highly densify biomass data on the condition that those are fused with future powerful long wavelength InSAR assets such as NISAR, TanDEM-L and Biomass monitoring mission for Carbon Assessment (BiOMASS) as shown in Qu et al. [30]. Finally, in addition to the improvement of land cover classification, the observations in our study, such as the temporal decay pattern of phase coherences, provides highly useful information to investigate stable scatterers in InSAR time series analysis. This is one of the most significant methods adopted in geodetic remote sensing, hence we will further extend to investigate the feasibility based on the time series InSAR packet approach demonstrated herein.

## Figures and Tables

**Table 1:** Information of ALOS PALSAR images and InSAR pairs covering Mt. Baekdu test area. Note that the empirical value of critical baseline for InSAR pair is 13 km; thus all InSAR pair of this study have proper conditions for InSAR phase coherence formation as shown in perpendicular baseline values.

Scene ID	Image Mode	Acquisition Time	Polarization	Perpendicular Baseline for InSAR (m)
ALPSRP185620830	FBD	07/19/2009 13:53	HH + HV	−509.31
ALPSRP192330830	FBD	09/03/2009 13:53	HH + HV	−504.51
ALPSRP199040830	FBD	10/19/2009 13:53	HH + HV	−553.58
ALPSRP212460830	FBS	01/19/2010 13:53	HH	−529.86
ALPSRP219170830	FBS	03/06/2010 13:53	HH	−288.15
ALPSRP225880830	FBS	04/21/2010 13:53	HH	−55.93
ALPSRP232590830	FBD	06/06/2010 13:52	HH + HV	−98.46
ALPSRP239300830	FBD	07/22/2010 13:52	HH + HV	−577.03
ALPSRP252720830	FBD	10/22/2010 13:51	HH + HV	−226.07
ALPSRP259430830	FBD	12/07/2010 13:50	HH + HV	−416.04
ALPSRP266140830	FBS	01/22/2011 13:49	HH	

**Table 2:** InSAR image information over Uljin test area.

Scene ID	Image Mode	Acquisition Time	Polarization	Perpendicular Baseline for InSAR (m)
ALPSRP091680720	FBD	10/14/2007 13:50	HH + HV	
ALPSRP105100720	FBS	01/14/2008 13:49	HH	503.168
ALPSRP111810720	FBS	02/29/2008 13:48	HH	596.462
ALPSRP125230720	FBS	05/31/2008 13:47	HH	165.874
ALPSRP131940720	FBD	07/16/2008 13:47	HH + HV	2952.705
ALPSRP138650720	FBS	08/31/2008 13:48	HH	2387.037
ALPSRP145360720	FBD	10/16/2008 13:48	HH + HV	940.708
ALPSRP152070720	FBS	12/01/2008 13:49	HH	143.166
ALPSRP158780720	FBS	01/16/2009 13:50	HH	545.277

**Table 3:** Processing information of Landsat and EO optical images which were employed for the validation.

Satellite	Processing Level	Resolution (m)	Orbit	Classification Method	Registration Accuracy	Acquisition Date
MODIS	MOD13	250		Monthly average of MODIS Terra		2009/09, 2010/07, 2011/01
EO-1 ALi (Baekdu)	Level 1Gst	30	Path: 116 Row: 30	SVM	>0.5 pixel accuracy with 11 check points	2008/08/30 02:00:15
Landsat 8 (Baekdu)	OLI/TIRS C1 Level-1	30	Path: 116 Row: 31	SVM	>0.5 pixel accuracy with 8 check points	2013/09/16 02:11:33
Landsat 8 (Uljin)	OLI/TIRS C1 Level-1	20	Path: 114 Row: 34	SVM	>0.5 pixel accuracy with 8 check points	2015-10-10 04:13:15

**Table 4:** Confusion matrix of PCA/ML and k-PCA/SVM classification scheme over Mt. Baekdu. Together with OV and KC.

PCA/ML	**Hh2**	**Water**	**Bare Field**	**Open/Mixed Forest**	**Conifer**	**Shrub/Grass**
	Water	0.886	0.071	0.017	0.021	0.006
	Bare field	0.072	0.463	0.278	0.155	0.017
	Open/mixed forest	0.000	0.000	0.126	0.874	0.000
	Conifer	0.002	0.010	0.373	0.585	0.001
	Shrub/Grass	0.035	0.391	0.343	0.221	0.010
	Water	OV = 0.983, KC = 0.373				
	Bare field	OV = 0.874, KC = 0.091				
	Open/mixed forest	OV = 0.845, KC = 0.414				
	Conifer	OV = 0.713, KC = −0.012				
	Shrub/Grass	OV = 0.824, KC = 0.255				
k-PCA/SVM	**HH2**	**Water**	**Bare Field**	**Open/Mixed Forest**	**Conifer**	**Shrub/Grass**
	Water	0.909	0.041	0.005	0.019	0.021
	Bare field	0.033	0.597	0.098	0.201	0.068
	Open/mixed forest	0.000	0.018	0.662	0.315	0.001
	Conifer	0.000	0.006	0.056	0.931	0.005
	Shrub/Grass	0.011	0.228	0.042	0.423	0.287
	Water	OV = 0.981, KC = 0.041				
	Bare field	OV = 0.830, KC = 0.135				
	Open/mixed forest	OV = 0.394, KC = 0.059				
	Conifer	OV = 0.500, KC = 0.177				
	Shrub/Grass	OV = 0.790, KC = 0.035				

**Table 5:** Confusion matrix of k-PCA/SVM classification scheme over Uljin.

**PCA**/**SVM**	**HH1**	**Farm-Land**	**Water**	**Artificial Structures**	**Mixed Forest**	**Broad-Leaf Forest**
	Farmland	0.29	0.02	0.28	0.26	0.15
	Water	0.00	0.99	0.01	0.00	0.00
	Artificial structures	0.11	0.04	0.44	0.30	0.10
	Mixed forest	0.02	0.00	0.06	0.61	0.32
	Broadleaf forest	0.01	0.00	0.04	0.37	0.58
k-**PCA**/**SVM**	**HH2**	**Farm-Land**	**Water**	**Artificial Structures**	**Mixed Forest**	**Broad-Leaf Forest**
	Farmland	0.25	0.02	0.30	0.14	0.28
	Water	0.00	0.99	0.01	0.00	0.00
	Artificial structures	0.11	0.04	0.37	0.29	0.18
	Mixed forest	0.02	0.00	0.14	0.55	0.29
	Broadleaf forest	0.05	0.00	0.13	0.19	0.63
